# Injectable adhesive carboxymethyl chitosan-based hydrogels with self-mending and antimicrobial features for the potential management of periodontal diseases†

**DOI:** 10.1039/d3ra00904a

**Published:** 2023-04-17

**Authors:** Xiaoqian Lin, Jia Lv, Desheng Wang, Kaikai Liu

**Affiliations:** a Department of Pharmacy, Qilu Hospital (Qingdao), Cheeloo College of Medicine, Shandong University Qingdao Shandong China; b Department of Prosthodontics, Qilu Hospital, Cheeloo College of Medicine, Institute of Stomatology, Shandong University Jinan Shandong China; c Jinan Stomatological Hospital Jinan Shandong China; d Department of Stomatology, Qilu Hospital (Qingdao), Cheeloo College of Medicine, Shandong University Qingdao Shandong China lkkqlyy@163.com

## Abstract

Treating periodontal diseases is a great challenge owing to the motion and wet conditions, bacterial infection, and tissue defects. Therefore, designing bioactive materials with outstanding wet-tissue adhesion, antimicrobial features, as well as favorable cell responses, is highly desirable to meet practical necessity. In this work, bio-multifunctional melatonin-loaded carboxymethyl chitosan/polyaldehyde dextran (CPM) hydrogels have been developed through the dynamic Schiff-base reaction. Our results demonstrate that the CPM hydrogels display injectability, structural stability, and high tissue adhesion in the wet and motional state, as well as self-healing features. In addition, the designed hydrogels show great antibacterial properties and excellent biocompatibility. The prepared hydrogels display a slow release of melatonin. Moreover, the *in vitro* cellular assay indicates that the developed hydrogels containing 10 mg per mL melatonin significantly promote cell migration. Thus, the synthesized bio-multifunctional hydrogels show great promise in the treatment of periodontal disease.

## Abstract 2

The CPM hydrogels fabricated in this work display injectability, structural stability, and high tissue adhesion in the wet and motional state, as well as self-healing features.

## Graphical abstract

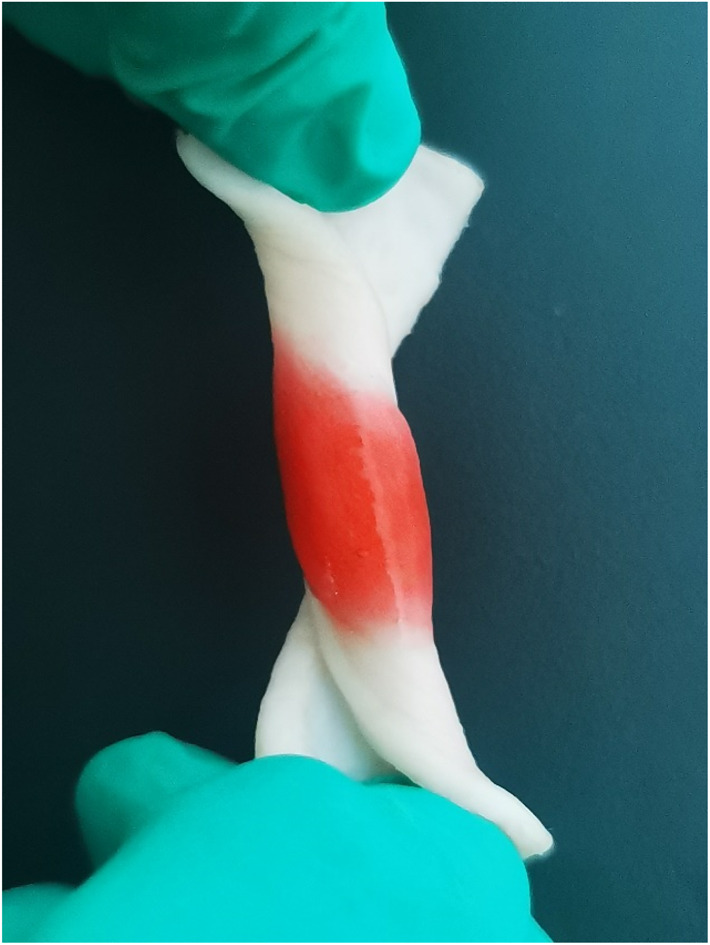

## 1. Introduction

Periodontal diseases are estimated to impact approximately 14% of the global adult population (more than 1 billion cases)^1^ and are mainly caused by physical trauma, bacterial infections, poor oral hygiene, as well as inflammation of the gums and bones.^2,3^ Particularly, periodontitis commonly results in redness, bleeding, swollen gums, bone resorption, imbalance of the oral flora, loss of teeth, and even other severe chronic diseases (*e.g.*, cardiovascular diseases, rheumatoid arthritis, Alzheimer's disease, chronic obstructive pulmonary disease, *etc.*).^4,5^ Clinical management is largely dependent on drugs delivered locally or orally. However, local drug delivery has several defects due to the flushes of saliva and movements, impacting their residence time and drug distribution.^6,7^ Additionally, oral drugs, when used for long time, cause significant side effects. Therefore, it is necessary to develop a novel biomaterial that can carry bioactive compounds, inhibit bacterial growth, and steadily integrate with the periodontium for a long time.

In recent years, multifunctional hydrogels obtained through the dynamic Schiff-base reaction have been demonstrated to possess several merits under physiological conditions, including injectability, structural stability, high wet-tissue adhesion, and self-healing characteristics, which have gained increasing attention in wound healing, tissue repair and regeneration applications.^8–19^ Generally, synthetic polymers are used to prepare the dynamic Schiff-base hydrogels, which lack excellent biocompatibility, abundant cell adhesion sites, and biofunctions. Carboxymethyl chitosan (CMCS), which contains amino groups, shows excellent biodegradability, biocompatibility, and anti-bacterial features.^15^ The oxidized dextran (PDA) with aldehyde groups can react with biomaterials containing amine groups to generate hydrogels for biomedical applications, such as tissue adhesives, smart drug delivery vehicles, and cell carriers. However, the CMCS/PDA (CP) composite hydrogels still lack specific bioactivity to treat periodontal disease. As reported, melatonin (MT), an endogenous indole hormone that exists in many organisms, such as bacteria, fungi, and mammals, possesses many biological activities, including tissue repair promotion, immunoregulation, antioxidant activity, and anti-tumor effects.^20,21^ Thus, CMCS/PDA/MT (CPM) composite hydrogels would be great candidates for the potential management of periodontal diseases.

Herein, bio-multifunctional CPM hydrogels with injectability, structural stability, high wet-tissue adhesion, and self-healing capacity were designed *via* the dynamic Schiff-base reaction. The physicochemical properties of these CPM hydrogels were characterized by Fourier transform-infrared (FTIR) spectroscopy, scanning electron microscopy (SEM), and so on. Their antibacterial activity and cytocompatibility were detected *in vitro*. The prepared CPM hydrogels have outstanding (bio)physicochemical characteristics for the potential management of periodontal disease compared with other hydrogel adhesives, as evidenced by the following observations: (1) their good injectability allows the hydrogels to enter the periodontal space. (2) One superior merit of the CPM hydrogels is their high wet-tissue adhesion, which could maintain stability during the motion of the oral cavity. (3) The CPM hydrogels possess excellent self-healing ability, which could prolong their life during mastication. (4) The CPM hydrogels display great antibacterial activity and enhance cell migration, which helps avoid tissue infection and promote tissue repair. This work suggests that the designed CPM hydrogels possess great potential for the management of periodontal diseases.

## 2. Materials and methods

### 2.1. The synthesis of PDA

The PDA prepared with a 1 : 1 molar ratio of dextran (Dex, *M*_w_ = 20 kDa, Shanghai Yuanye Biotechnology Co., Ltd.) and sodium periodate (NaIO_4_, Sinopharm Chemical Reagent Co. Ltd., China) was named PDA50. Dex and NaIO_4_ were dissolved in deionized water and stirred for 24 h without light at room temperature. Then, the solution was dialyzed (molecular weight 8–10 kDa, Shanghai Yuanye Bio-Technology Co. Ltd., China) with single-distilled water, during which the dialysate was changed frequently. The final solution obtained after dialysis was freeze-dried (Alpha 1-2 LDplus; CHRIST®, Germany) to get a white solid.

### 2.2. Preparation of the CPM hydrogels

The CP hydrogels were fabricated by mixing an equal volume of 10% CMCS/phosphate-buffered saline (PBS) and 10% PDA50/PBS solutions. MT (Aladdin Biological Reagent Co., Ltd.) was added to obtain CPM hydrogels containing 5 and 10 mg per mL MT.

### 2.3. Physicochemical characterization

The FTIR spectra of the samples were obtained using a Nicolet iN10 FTIR spectrometer (Thermo Fisher Scientific, Waltham, MA, USA) at 2 cm^−1^ resolution over 32 scans.

The ^1^H NMR spectra (Bruker Advance III, German) of the samples were detected at room temperature using D_2_O as the solvent.

The dried samples were imaged by an SEM (VEGA3, TESCAN, Czech) with an acceleration voltage of 10 kV. The pore size of the samples was obtained by Image J software.

### 2.4. Swelling ratio calculation

The dry hydrogel samples were initially weighed (*M*_0_) and then immersed in PBS at 37 °C. They were taken out at specified time intervals from PBS and weighed (*M*_s_).
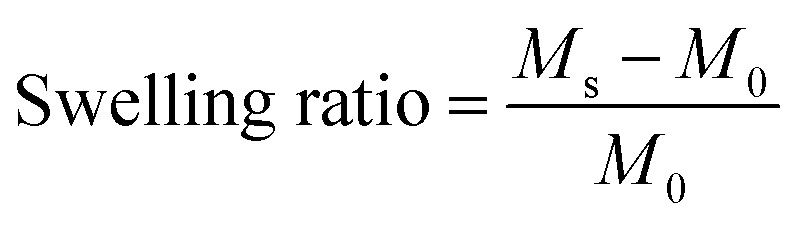


### 2.5. Self-mending feature assessment

CPM hydrogels with various colors were injected into “T” shape molds. The shaped hydrogel was cut into two pieces, and subsequently, the separated pieces were put together. Their self-mending ability was photographed.

### 2.6. Adhesion assessment

The samples were applied to the pigskin surface, and the skin specimens were twisted and pulled to evaluate their adhesion properties.

### 2.7. *In vitro* MT release of the CPM hydrogels

To further simulate the infected oral microenvironment, the CPM hydrogels were placed in dialysis bags (molecular weight 3500 Da) containing a PBS (pH = 7 or 5) buffer solution. The hydrogels in the dialysis bags were oscillated in centrifuge tubes containing 20 mL PBS buffer solution at 37 °C. The dialyzed solution was taken out at regular intervals, fresh PBS buffer was added and shaking at 37 °C was continued. The UV absorption of MT in the dialyzed solution was detected at 278 nm using an ultraviolet spectrophotometer.

### 2.8. Evaluation of the antimicrobial activity

To test the antimicrobial effect of the hydrogels, *S. aureus* and *E. coli* were selected as model bacteria. Along with blank and CP as the control groups, the CPM hydrogels measuring a total volume of 200 μL were prepared, and irradiated by ultraviolet radiation for 30 min on a super-clean table. The hydrogels were placed in the tryptone soybean broth of *S. aureus* or the Luria–Bertani medium of *E. coli* at the same concentration, and the bacteria were cultured in the incubator at 37 °C for 12 h. Each co-cultured bacterial solution was diluted 10^−8^ times with PBS (pH = 7), and 20 μL PBS-diluted bacterial broths were evenly smeared on agar plates, which were placed in the incubator at 37 °C. After 24 hours, the number of colonies on the agar medium (CFU) was observed, and each sample was grown on 3 plates. The antimicrobial activity of the hydrogels was computed as follows:
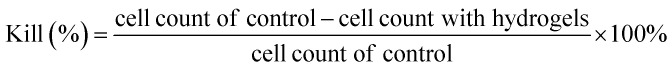


### 2.9. The cytocompatibility test

L929 mouse fibroblasts (Shanghai Institutes for Biological Sciences, Chinese Academy of Sciences, China) were cultured in the DMEM cell medium (Biological Industries) with 10% fetal bovine serum (FBS) and 1% penicillin/streptomycin in a CO_2_ incubator at 37 °C. During cell passage, the cells were generally digested for 3 min with 0.05% trypsin/EDTA.

The L929 cells were seeded on a 96-well plate at 5 × 10^3^ cells per well and cultured in a CO_2_ incubator. After 24 hours, the hydrogel extract was co-cultured with the L929 cells for 24, 48, and 72 h. Then, 10 μL CCK-8 (Sigma-Aldrich) solution was added to each well. The absorbance (OD) of CCK-8 was detected on a microplate reader.

The L929 cells were seeded in a 24-well plate at the density of 2 × 10^4^ cells per well. After 24 hours, the hydrogel extracts were added and incubated for 24 h. PI and calcein-AM (Dalian Meilun Biotechnology Co., Ltd.) diluted with PBS were added and incubated for 20 min, and the cells were photographed using a fluorescence microscope.

### 2.10. Cell migration

For the cell migration assay, 2 × 10^5^ cells per well were seeded in a 6-well plate. The cells in each well were scratched with a 200 μL pipette tip after 24 h to form gaps ∼400 μm in width, and washed with PBS three times. Different serum-free hydrogel extracts were added and incubated for 24 h. The complete DMEM medium was added next and incubated for 0, 12, 24 and 48 h, and the cell layers were photographed under a fluorescence microscope.

## 3. Results and discussion

### 3.1. Fabrication and characterization of the CPM hydrogels

**Fig. 1:**
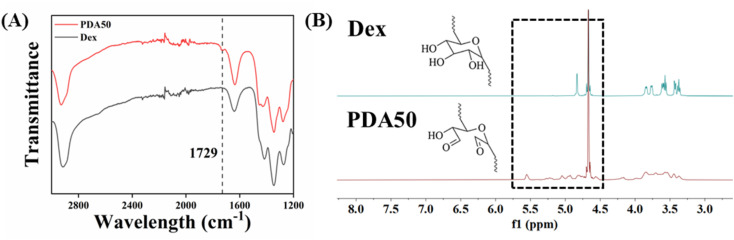
(A) The FTIR and (B) ^1^H NMR spectra of Dex and PDA.

To test whether PDA was synthesized, FT-IR and ^1^H NMR were carried out. Fig. 1A shows that a carbonyl stretching peak corresponding to the aldehyde group was found at 1730 cm^−1^ in the FTIR spectra.^22^Fig. 1B displays several new peaks in the spectrum of PDA at 4.0–5.6 ppm compared with Dex.^22^ The peaks at 4.8–5.6 ppm were assigned to the hemiacetal groups; as confirmed by ^1^H NMR under alkaline conditions (pH = 13), hemiacetal decomposed back to aldehyde, with the disappearance of peaks at 4.8–5.6 ppm and the generation of characteristic aldehyde signals at 8.30 ppm (Fig. S1†).^23^ All the above data suggest that PDA was synthesized successfully. In addition, the oxidative degree of PDA was identified by the colorimetric method using hydroxylamine hydrochloride. When the molar ratio of Dex to NaIO_4_ was 2, aldehyde formation was 23.04%.

**Fig. 2:**
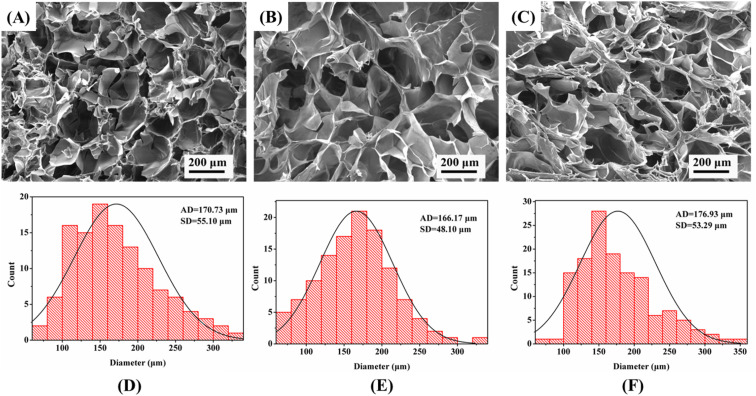
The SEM images of the (A) CP, (B) CPM1, and CPM2 hydrogels and (D–F) their corresponding pore size distributions.

SEM was carried out to assess the microstructure of the prepared hydrogels. It was found that the injectable CP-based hydrogels (Fig. S2†) showed a 3D, relatively uniform, and interconnected pore morphology, indicating their homogeneous microscopic structure and chemical feature (Fig. 2A–C). Fig. 2D displays that the pore sizes of CP, CPM1, and CPM2 hydrogels were 170 ± 55, 166 ± 48, and 177 ± 53 μm, respectively. There were no significant differences among them. The presence of larger pores is favorable for drug release and other biomedical applications.^24,25^

**Fig. 3:**
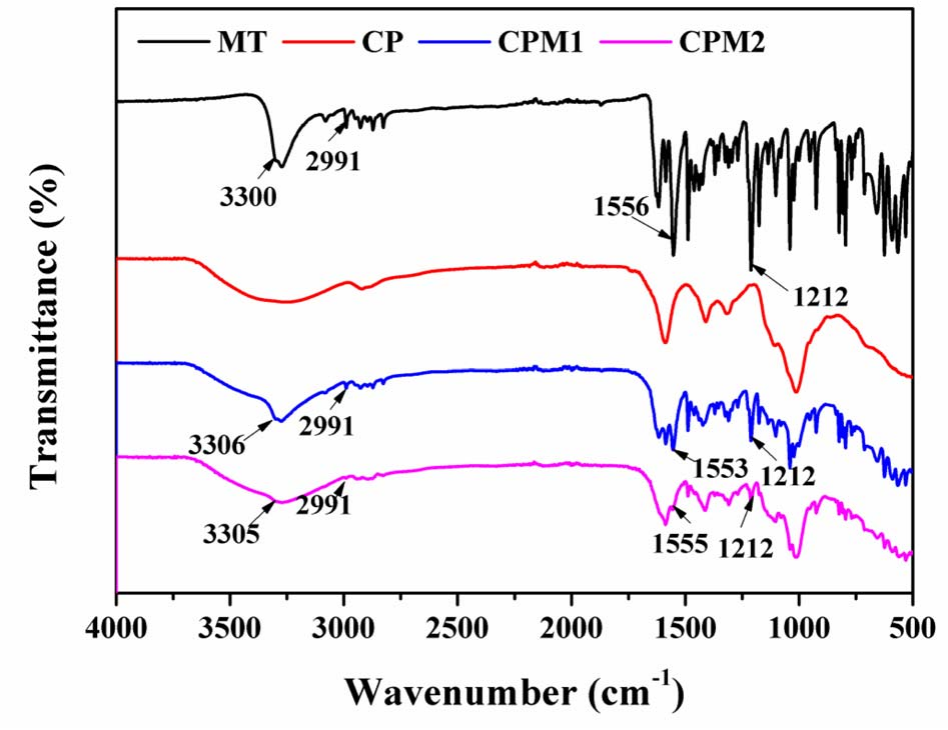
The FTIR spectra of melatonin (MT), the CMCS/PDA hydrogel (CP), and the CMCS/PDA hydrogels containing 5 mg per mL MT (CPM1) and 10 mg per mL MT (CPM2).

The chemical features of the CPM hydrogels were characterized by FTIR. The chemical features characterized by FTIR confirmed the successful synthesis of the CMCS/PD hydrogels, as the carbonyl stretching peak (1732 cm^−1^) from the aldehyde groups disappeared in the CMCS/PD hydrogels, while a new peak at 1643 cm^−1^ was detected, indicating the formation of the imine structure (–HC=N–). In Fig. 3, the infrared characteristic absorption peaks of MT show the stretching vibration of N–H at 3300 cm^−1^, the stretching vibration of saturated –CN at 2991 cm^−1^, the stretching vibration of –CN in the secondary amide in the molecular structure of MT at 1556 cm^−1^, and the stretching vibration of aromatic ether C–O–C at 1213 cm^−1^.^26–28^ Between 3000 and 3500 cm^−1^, the peaks of CPM1 and CPM2 hydrogels were stronger than those of CP, indicating the increase in N–H bonds in CPM. Compared with the infrared absorption peak of CP, there was an obvious stretching vibration characteristic of saturated C–H in both CPM1 and CPM2 hydrogels at 2991 cm^−1^, the C–O–C stretching vibration of aromatic ether at 1212 cm^−1^, a characteristic absorption peak of CPM1 at 1553 cm^−1^, and the CN stretching vibration of the secondary amide at 1555 cm^−1^. Therefore, the characteristic peaks of MT appeared in both CPM1 and CPM2 hydrogels. This shows that MT was loaded in both CP hydrogels.

**Fig. 4:**
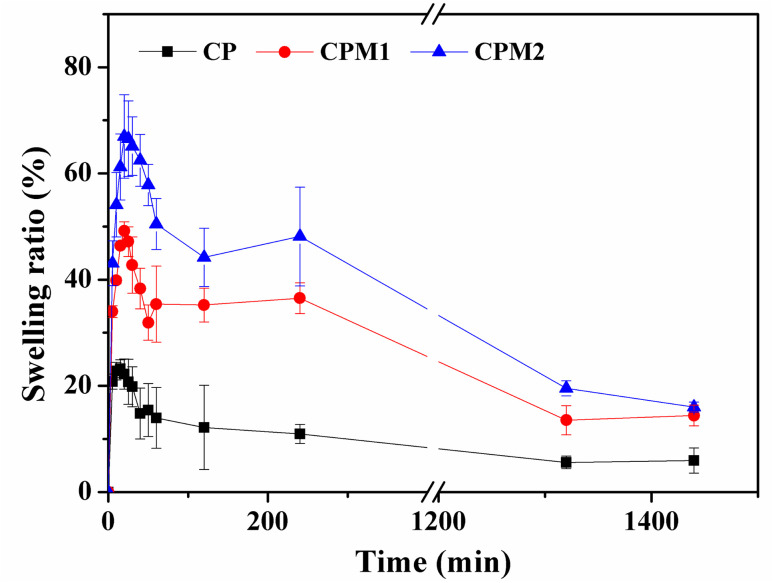
The swelling ratios of the lyophilized hydrogels.

The swelling feature of the biomaterial is a critical parameter considered for its application in the treatment of periodontal disease. Swelling does not only weaken the hydrogel, but also can damage surrounding tissues or delay their healing. Based on the swelling test of the hydrogels, as shown in Fig. 4, the CP, CPM1 and CPM2 hydrogels exhibited two swelling processes in the PBS solution, and the swelling rate of hydrogels reached the highest at 30 min, which is due to their larger pore size and faster water absorption. After 30 min, the weight of the three hydrogels began to decrease. When the swelling reached saturation, it was found that their weights had decreased compared with the initial values, probably because of the degradation of the hydrogels. When the hydrogels were in PBS for 24 h, the swelling process basically reached a dynamic equilibrium. In PBS solution, within 24 hours, the shape of the hydrogels did not change, and the structure remained stable.

### 3.2. Self-healing and wet-tissue adhesion tests of the hydrogels

**Fig. 5:**
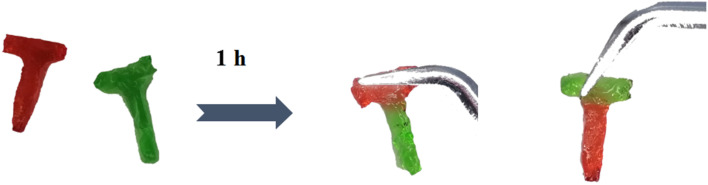
Photos of the self-mending process of the CPM hydrogel.

When used for the treatment of periodontitis, the hydrogel would inevitably receive external stress, resulting in fracture and shortening their life.^29^ If the hydrogel has self-healing properties, it can maintain structural integrity, restore its original function, and prolong service life through self-repair. As shown in Fig. 5, two colored “T” samples were cut in half, and the halves of different colors were spliced together and kept for 1 h. When the new spliced “T” samples were lifted by forceps, they did not fall off, suggesting the excellent self-mending feature of the CPM sample.

**Fig. 6:**
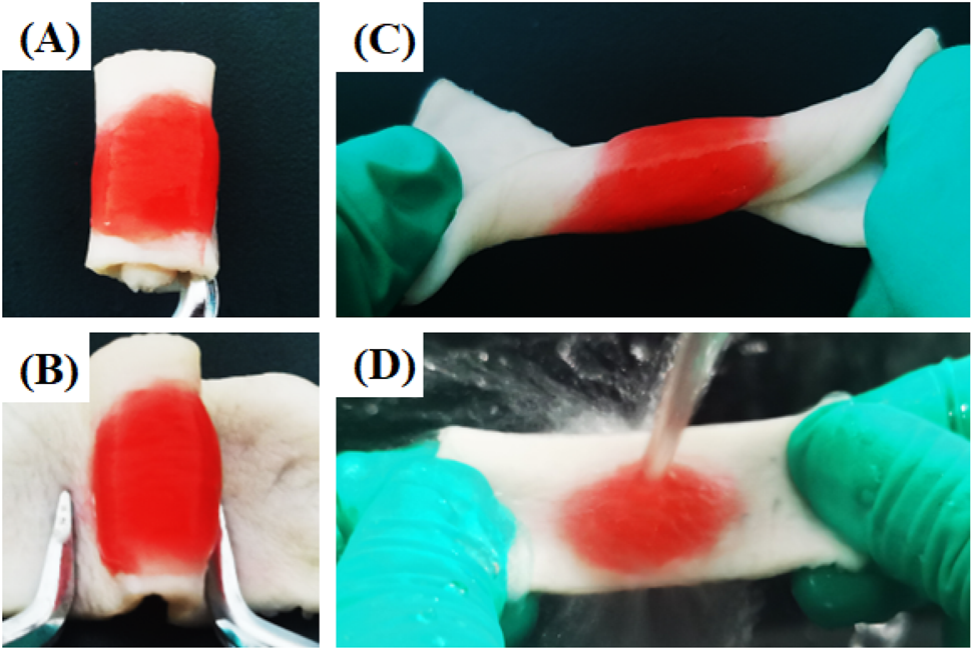
(A–C) Photos of the wet-tissue adhesion of the CPM hydrogels in different twisted states on pigskin. (D) Photos of the CPM hydrogels on the surface of pigskin under the impact of water flow.

The wet-tissue adhesion of the hydrogel is critical in an oral environment with the flushes of saliva and motion of the oral cavity to ensure stable integration with the periodontium for a long time.^30–32^ To demonstrate the wet-tissue adhesion property of the designed samples, the CPM sample was injected into the interface of wet pigskin *via* a double mixed needle and stayed strongly adhered to the tissue surface when twisted in various manners (Fig. 6). This shows that CPM hydrogel not only firmly adhered to the tissue, but also had a certain softness and could be crimped. When placed under running water, the hydrogel on the surface of the pigskin remained adhered firmly to the pigskin. These results demonstrate that the CPM hydrogel exhibited great wet-tissue adhesion ability for application in the periodontium environment.

### 3.3. *In vitro* MT release from the hydrogels

**Fig. 7:**
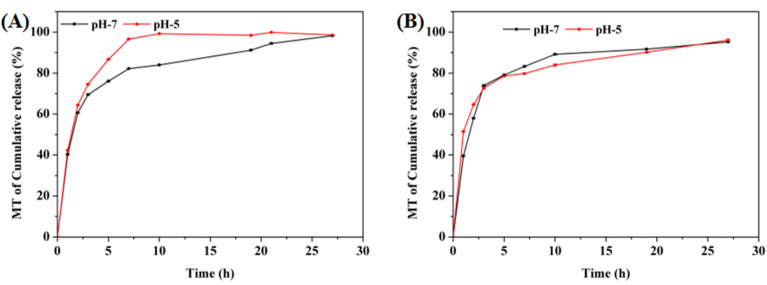
The MT release curves of the (A) CPM1 and (B) CPM2 hydrogels in PBS (pH = 5 and 7).

To verify MT release from the CPM hydrogels *in vitro*, the samples were placed in a simulated periodontal environment, and the content of MT in the solution was tested at different times. As shown in Fig. 7, the CPM1 and CPM2 hydrogels were placed in PBS (pH = 5 and 7), and they released more than 90% MT into their environments within 24 h; 98% MT was released by CPM1 in PBS (pH = 5).

### 3.4. Evaluation of the antibacterial activity

**Fig. 8:**
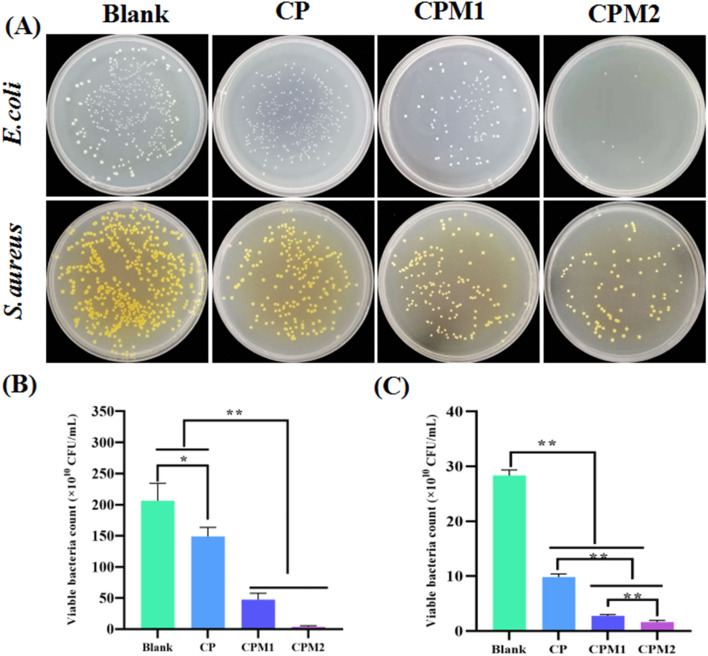
(A) Photos of the viable bacterial colonies on agar plates after co-culture with the hydrogels. (B) CFU of *S. aureus* after treatment with hydrogels. (C) CFU of *E. coli* after treatment with hydrogels. The data represent the mean ± SD (*n* = 3; *p < 0.05, **p < 0.01).

Most periodontal diseases, particularly periodontitis, are mainly caused by bacterial infections.^33,34^ Through *in vitro* antibacterial assays, the antibacterial ability of the CP-based hydrogels was verified based on bacterial CFUs after treatment. As shown in Fig. 8A, the CP hydrogels displayed significantly antibacterial effect on *E. coli* and *S. aureus* compared with the blank group. When loaded with MT, the number of bacteria reduced greatly after CPM1 and CPM2 were co-cultured with *E. coli* and *S. aureus*. In Fig. 8B, the average number of *E. coli* colonies in the blank group was 2.06 × 10^12^ CFU mL^−1^, while those in the CPM1 and CPM2 groups were 4.75 × 10^11^ CFU mL^−1^ and 3.6 × 10^8^ CFU mL^−1^, respectively. The average colony number of *S. aureus* in the blank group was 2.84 × 10^11^ CFU mL^−1^, while those in the CPM1 and CPM2 groups were 2.79 × 10^10^ and 1.65 × 10^10^ CFU mL^−1^, respectively (Fig. 8C). From the above results, the CPM hydrogel possesses great antibacterial effect, probably because (i) the aldehyde groups in the hydrogel interact with the primary amine groups on the bacterial membrane, thus suppressing bacterial growth; (ii) the cationic amine groups of CMCS interact with the negative-charged bacteria, thereby penetrating the bacterial membrane and causing the leakage of the intracellular fluid.

### 3.5. Cytocompatibility and cell migration assessment

**Fig. 9:**
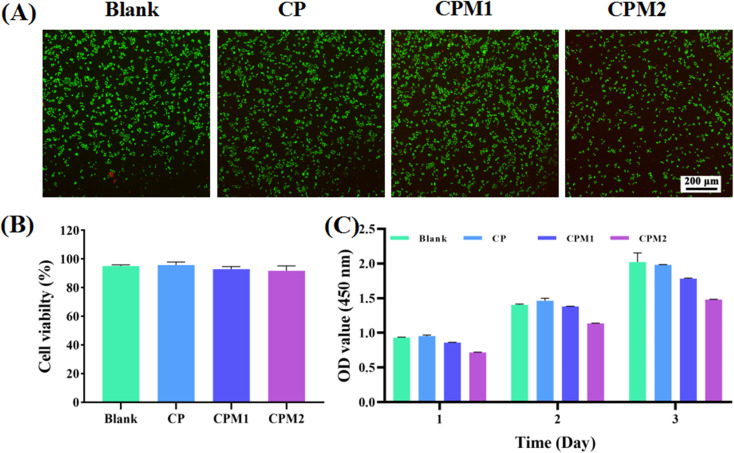
(A) The live/dead fluorescence images and (B) living cell percentage of L929 cells co-cultured with the hydrogel samples for 24 h. (C) The OD value of the L929 cells incubated with the hydrogels for 1, 2 and 3 d. The data represent the mean ± SD (*n* = 3).

The assessment of cytocompatibility is critical to demonstrate the suitability of the hydrogel for the treatment of periodontal diseases.^35,36^ CCK-8 measurement and live/dead staining were performed to test the interaction between the hydrogels and L929 mouse fibroblasts. As shown in Fig. 9, the fluorescence images after live/dead staining were obtained after co-culturing the cells with different hydrogels for 24 h. The living/dead cells were marked with calcein-AM (green) and PI (red), respectively. It was found that CP, CPM1 and CPM2 had no toxicity toward the L929 cells. In Fig. 9B, the living cell percentage after treatment with the samples is further analyzed. There was no significant difference in the proportion of living cells among the different groups. The results show that CP, CPM1 and CPM2 display excellent cytocompatibility. Furthermore, the proliferation of L929 cells induced by the three hydrogels was detected by CCK-8. As shown in Fig. 9C, the number of L929 cells in each group increased with the extension of culture time after 1, 2 and 3 d of co-culture with the hydrogels. Taken together, the data imply that the CPM hydrogels have acceptable cytocompatibility for periodontal diseases.

**Fig. 10:**
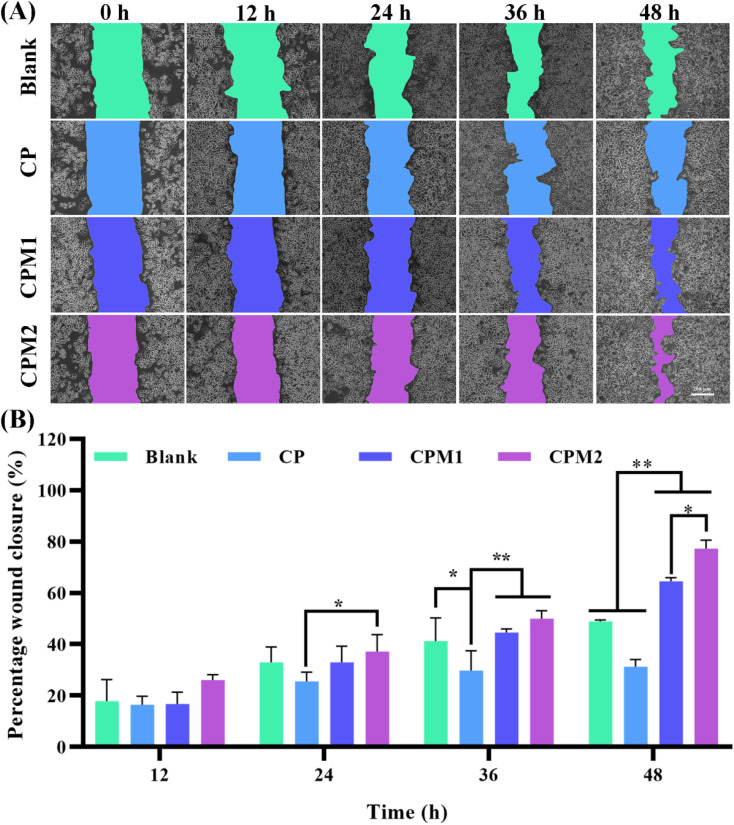
(A) The representative images of L929 cell migration from the scratch healing assay at different times. (B) Percentage wound closure at 0, 12, 24, 36 and 48 h when treated with the hydrogels. The data represent the average ± SD (*n* = 3) (*p < 0.05, **p < 0.005).

A cell scratch test was used to detect the impact of the prepared samples on cell migration. As shown in Fig. 10A, the CP-based hydrogels co-incubated with L929 cells for 24 h had a slight effect. After 36 h of incubation, compared with the control group, cell migration began to occur in the CPM1 and CPM2 groups, and the scratch area healing rate of the CPM1 group was 45% (Fig. 10B). The scratch area healing rate of the CPM2 group was more than 50%. After 48 h, the healing rate of the scratch area in the CP group was 32%, which was lower than that in the blank group (49%). However, in the presence of MT-loaded CP hydrogel, there were largely migrating cells in the scratched part, and the cell migration rate in the CPM2 group reached 79%. Therefore, CPM hydrogels can significantly enhance cell migration, which can accelerate periodontal repair.

## 4. Conclusions

In summary, multi-biofunctional MT-loaded CP hydrogels were fabricated *via* the dynamic Schiff base reaction. These CPM hydrogels possessed injectability, wet-tissue adhesion, as well as self-healing characteristics. Furthermore, the developed hydrogels exhibited excellent antibacterial ability and cytocompatibility. The design hydrogels showed a slow release of MT. In addition, the cellular experiments suggest that the CPM2 hydrogels can largely enhance cell migration. Therefore, the fabricated bio-multifunctional hydrogels display great application potential in the management of periodontal diseases. In addition, we expect that the strategy employed in this work for the preparation of injectable adhesive carboxymethyl chitosan-based hydrogels with self-mending and antimicrobial features is flexible and can be extended to achieve wet-tissue adhesive drug delivery for the treatment of other oral diseases.

## Conflicts of interest

The authors declare no competing financial interest.

## Supplementary Material

RA-013-D3RA00904A-s001
