# Supplementary material for: Injectable adhesive carboxymethyl chitosan-based hydrogels with self-mending and antimicrobial features for the potential management of periodontal diseases
Source: RSC Adv. 2023 Apr 17;13(18):11903–11. doi: 10.1039/d3ra00904a (PMC10107338; doi:10.1039/d3ra00904a)
Supplement: RA-013-D3RA00904A-s001 [file RA-013-D3RA00904A-s001.pdf]

Electronic Supplementary Material (ESI) for RSC Advances.
This journal is © The Royal Society of Chemistry 2023

Injectable Adhesive Carboxymethyl Chitosan-based Hydrogels with Self-mending and Antimicrobial Features for the Potential Management of Periodontal Disease

Xiaoqian Lin[1], Jia Lv[2]， Desheng Wang[3], Kaikai Liu *[4]

Author Affiliations:

[1] Department of Pharmacy, Qilu Hospital (Qingdao), Cheeloo College of Medicine, Shandong University, Qingdao, Shandong, China

[2] Department of Prosthodontics, Qilu Hospital, Cheeloo College of Medicine, and Institute of Stomatology, Shandong University, Jinan, Shandong, China

[3] Jinan Stomatological Hospital, Jinan, Shandong, China.

[4] Department of Stomatology, Qilu Hospital (Qingdao), Cheeloo College of Medicine, Shandong University, Qingdao, Shandong, China

*Corresponding author:

Kaikai Liu, Email: lkkqlyy@163.com

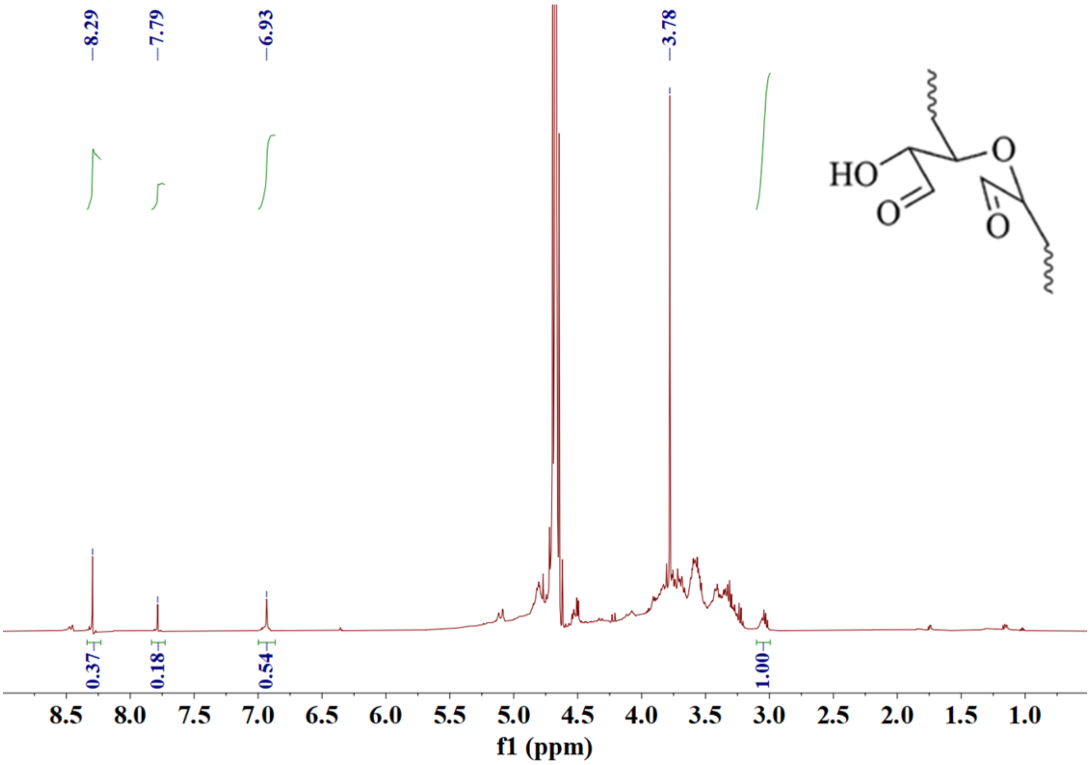

Figure S1. ^{1}H NMR spectra of PDA under alkaline condition (pH=13).

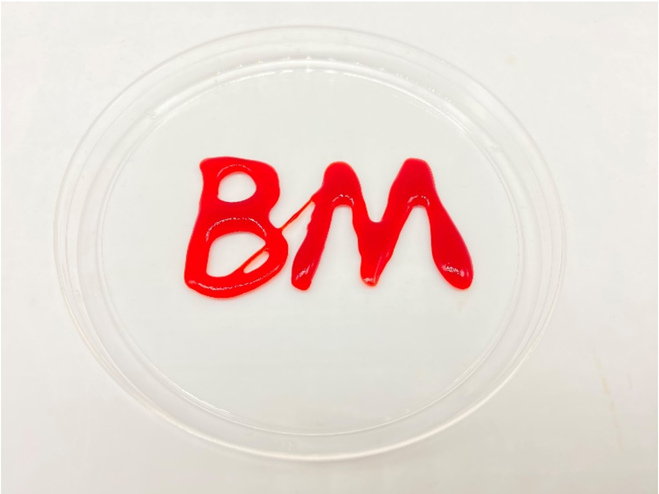

Figure S2. Injectable ability of CPM hydrogels.
